# Diversity and Representation in Cardiovascular Research: Evidence Gaps, Emerging Models, and Policy Implications

**DOI:** 10.3390/ijerph23020241

**Published:** 2026-02-14

**Authors:** Simran Grewal, James Wildish, Catherine Chalmers, Christine Dedding, Jeanine Suurmond, Charles Agyemang, Nimrat Grewal

**Affiliations:** 1 Shoulder and Elbow Unit, Department of Orthopaedic Surgery, Onze Lieve Vrouwe Gasthuis, 1091 AC Amsterdam, The Netherlands; s.k.grewal@olvg.nl; 2 Department of Research, The Original Fit Factory, Glasgow G62 7LW, UK; 3 Department of Ethics, Law & Humanities, F-Vleugel Medische Faculteit, Amsterdam UMC, De Boelelaan 1089a, 1081 HV Amsterdam, The Netherlands; c.dedding@amsterdamumc.nl; 4 Department of Primary and Community Care, Radboudumc, 6525 GA Nijmegen, The Netherlands; 5 Department of Public and Occupational Health, Amsterdam Public Health, University of Amsterdam Medical Centers, 1105 AZ Amsterdam, The Netherlands; c.o.agyemang@amsterdamumc.nl; 6 Division of Endocrinology, Diabetes, and Metabolism, Department of Medicine, Johns Hopkins University School of Medicine, Baltimore, MD 21205, USA; 7 Department of Cardiothoracic Surgery, Amsterdam University Medical Center, Location AMC, 1105 AZ Amsterdam, The Netherlands; 8 Department of Cardiothoracic Surgery, Yale New Haven Hospital, Yale University School of Medicine, New Haven, CT 06510, USA

**Keywords:** health equity, cardiovascular disease, data diversity, citizen science, community-based research, global health

## Abstract

**Highlights:**

**Public health relevance—How does this work relate to a public health issue?**

- Cardiovascular disease guidelines are largely based on homogeneous datasets, limiting their validity for women, ethnic minorities, and populations outside HICs.
- Structural under-representation in cardiovascular research contributes directly to biased risk prediction, delayed diagnosis, and unequal health outcomes worldwide.

**Public health significance—Why is this work of significance to public health?**

- This work reframes diversity in cardiovascular data as a scientific necessity for accurate, generalizable, and effective prevention and treatment strategies.
- It synthesizes evidence gaps and emerging global models (e.g., data donation, inclusive biobanking, participatory research) into a coherent roadmap for equitable cardiovascular science.

**Public health implications—What are the key implications or messages for practitioners, policymakers and/or researchers in public health?**

- Researchers and regulators must embed diversity, data sovereignty, and community governance into study design, data infrastructures, and guideline development.
- Policymakers and clinicians should move from “one-size-fits-all” cardiovascular guidelines toward context-sensitive, population-aware risk models and prevention strategies.

**Abstract:**

Although cardiovascular disease (CVD) is the leading cause of mortality globally, it remains insufficiently understood in large parts of the world. The scientific foundations underpinning CVD risk prediction, diagnostics, and treatment are extensively derived from homogenous datasets, primarily including White, male participants from high-income countries. This lack of diversity and inclusion can lead to biased evidence, which in turn contributes to reduced diagnostic accuracy and the under-representation of key populations, and ultimately limits the generalizability of trial results and guidelines. In this paper, we discuss that diversity in cardiovascular data is a scientific necessity for valid and globally applicable knowledge and not just a matter of fairness. Drawing from emerging initiatives in genomics, digital health, and participatory research, we propose a global roadmap to reshape how cardiovascular research is conducted. This includes strategies such as data donation frameworks, inclusive biobanking, equitable AI development, and international policy change. Only by integrating diversity into scientific methodologies can we ensure that cardiovascular guidelines are effective, inclusive, and just.

## 1. Introduction

Cardiovascular disease (CVD) is the world’s leading cause of death, accounting for an estimated 18 million lives annually [1]. Traditionally, CVD was considered a disease of affluence; however, recent global data indicate a marked shift in this pattern. Over three-quarters of CVD-related deaths now occur in low- and middle-income countries (LMICs) [2]. Yet the data and evidence that guide prevention, diagnosis, and treatment are disproportionately based on a narrow subset of the global population: predominantly White, male individuals from high-income countries (HICs) [3,4,5]. From randomized clinical trials to genetic studies and digital health algorithms, most large-scale cardiovascular research has focused on participants who do not reflect the demographic and genetic diversity of the world’s population. Illustratively, Sardar et al. showed that women, elderly patients, and those of non-White racial backgrounds were under-represented in the RCTs of the ACC/AHA guidelines for atrial, heart failure and unstable angina/non-ST-segment myocardial elevation [6]. These findings raise concerns about clinical trial enrollment and the generalizability of the guidelines in these under-represented populations.

The consequences of this biased foundation are wide-ranging and deeply consequential. For instance, polygenic risk scores for coronary artery disease aneurysm have been primarily developed using European-ancestry cohorts [7,8]. When applied to individuals of African, South Asian, or Indigenous descent, their predictive accuracy sharply declines [9,10,11,12,13]. Risk models and guideline thresholds based on these scores risk underestimating disease burden or delaying intervention in non-European populations [9,10,11,12,13]. Likewise, guideline-recommended surgical thresholds for aortic repair, anchored in absolute aortic diameters, do not sufficiently account for population-level differences in body size, aortic dimensions, or tissue pathology, particularly in women and non-Western populations [14,15].

Sex-based disparities further complicate the generalizability of evidence-based clinical guidelines. Women are often under-represented in cardiovascular trials, and sex-specific findings, when reported, are rarely powered to inform clinical decisions [16]. An analysis of 740 completed cardiovascular trials involving 862,652 participants revealed that only 38.2% of the study population were women [16]. And even when women are included in clinical studies, the impact of sex and ethnicity on health outcomes is often overlooked or insufficiently reported [17]. Differences in symptom presentation, drug metabolism, and anatomical characteristics are frequently overlooked, leading to delays in diagnosis and suboptimal outcomes for female patients [18,19].

These disparities do not result from sampling variability; they are the result of a structural pattern in science. Biomedical research infrastructures, funding mechanisms, and academic priorities have long prioritized institutions in HICs, often at the expense of LMICs-led research [20,21,22].

Language barriers, digital exclusion, lack of access to ethical frameworks, and historical mistrust in healthcare systems all limit the participation of diverse populations [23,24]. In many cases, individuals are either unaware of ongoing studies or are not invited to participate, even when they are most affected by the disease being studied.

In this paper, we focus on the current evidence-based medicine in cardiovascular science, built on extrapolation from narrow population heterogeneity. There is a lack of evidence to support inclusive, globally usable guidelines. To support truly inclusive and effective care, we must reconsider not only what data is collected, but how, where, and with whom we conduct research. Diversity in science is not a secondary goal or political gesture; it is essential to the validity, accuracy, and applicability of evidence-based medicine.

In the sections that follow, we examine the magnitude and mechanisms of the diversity gap in cardiovascular science, highlight emerging models of inclusive data generation and data donation, and propose a structured global roadmap to embed equity in cardiovascular guidelines. This paper is written as a narrative review. We draw on literature from cardiovascular research, genomics, digital health, participatory research, and policy to bring together evidence that is usually discussed in separate domains. Our selection of studies was guided by their relevance to the central question of this review: how a lack of diversity in cardiovascular data affects risk prediction, guidelines, and clinical care, and which emerging models may help address these gaps.

In this review, race is defined as a social construct reflecting lived experiences of structural inequity and a biological category. Ethnicity refers to shared cultural, linguistic, or ancestral background, while ancestry is used to denote genetic lineage. Data sovereignty is used to describe frameworks in which individuals or communities retain authority over how data are collected, governed, and used.

## 2. Pathways to Inclusive Evidence Generation

Though it is urgently needed, there is a significant gap in comprehensive data among populations that could explain local healthcare delivery patterns and patient outcomes. This gap is primarily evident when it comes to addressing disparities within minority populations, where studies have repeatedly shown significant higher readmission rates among minorities compared to White individuals. These differences are primarily driven by socioeconomic, literacy, and comorbidity disparities. Building equitable cardiovascular science requires acknowledgment of structural exclusion as well as concrete methods for change. The following section explores emerging strategies to diversify data, decentralize participation, and ensure fair representation in knowledge generation.

### 2.1. Diversify Data

**Figure 1:**
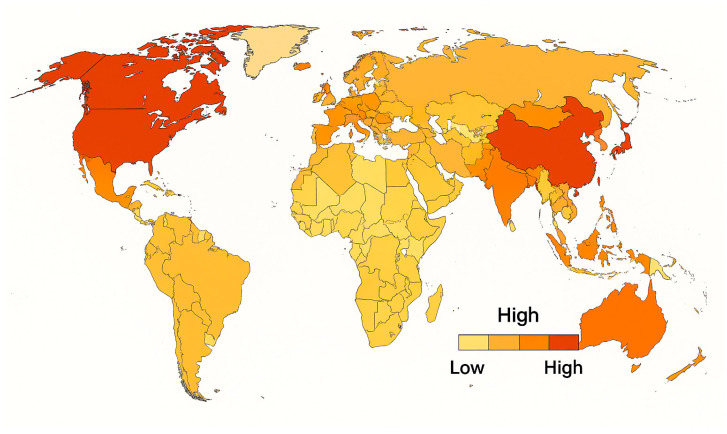
Global distribution of biomedical research output. This choropleth map shows the relative contribution of countries to high-impact biomedical research publications, with color intensity indicating publication volume. Color gradations reflect relative output and do not account for research quality, citation impact, or funding per capita.

During the 19th century, Western science gained prominence in part due to Europe’s military and technological dominance [20]. In the present day, biomedical research and innovation are largely concentrated within the world’s leading economies. The United States and China alone account for nearly 60% of all publications in high-impact health science journals, with additional contributions (approximately 22%) from countries such as Germany, the United Kingdom, Japan, France, and South Korea [21] (Figure 1). These nations not only have high gross domestic product levels, but also prioritize investment in biomedical research and maintain robust infrastructures that foster sustained scientific advancement [22].

The accessibility and convenience of academic centers, which are often located in high-income, urban regions of a country, have thus historically shaped the field of cardiovascular research. As a result, over the years, studies have disproportionately sampled cohorts from populations with higher socioeconomic status, greater health literacy, and easier access to tertiary care [25]. This type of bias is called *urban sampling*, and it inherently excludes large swaths of the global population, including rural communities, ethnic minorities, migrants, and individuals with lower education or digital skills [25].

As a result, a transformative methodology called *community-based participatory research* (CBPR) has been developed [26,27]. CBPR highlights building collaborative partnerships in which community members and stakeholders are equally involved at every stage of the research process, from planning and implementation to evaluation and dissemination. The *Jackson Heart Study*, for example, involved one of the largest cardiovascular cohorts focused exclusively on African Americans. In this study, the participants were not merely subjects, but co-creators; they were involved in the design, implementation, and dissemination of findings [28,29]. This approach increased both trust and retention, while generating insights directly relevant to the population at hand [30].

Other examples of CBPR include tele-cardiology projects in India, where local health workers operate mobile ECG and echocardiography units to assess rural populations [31,32]. In Brazil’s urban favelas, community health agents have been trained to identify early signs of hypertensive heart disease and facilitate enrollment into mobile monitoring programs [33,34]. In sub-Saharan Africa, mobile units equipped with point-of-care diagnostics help collect population-specific data on hypertension and valvular disease [35]. Several cardiovascular screening programs in Indigenous communities in Canada are co-led by tribal health councils [36,37]. These cooperative programs underscore the importance of embedded research models that work *with* communities rather than *on* them, ensure that screening is not only clinically effective but also culturally sensitive [36,37].

Moreover, when considering diversity, intersectional approaches which account for overlapping axes of identity, such as gender, ethnicity, age, and disability, are essential. For instance, women of reproductive age in some cultures may be excluded from studies due to norms around mobility or consent [38,39]. Older adults with cognitive impairment are often purposefully systematically excluded from research that does not specifically target them based on rigid consent procedures [40]. An intentional study design and culturally competent research teams who understand the social and ethical nuances of inclusion are mandatory to address these gaps.

Critically, this also requires a shift in governance models. The principle of data sovereignty, which is the right of communities to determine how their health data are collected, stored, and used, must become central. Especially in LMICs and Indigenous contexts, historical practices of data extraction without local benefit have eroded trust [41]. To address this, equitable research should be grounded in shared governance structures, such as locally defined data-use agreements, community representation in oversight bodies, and mechanisms that ensure reciprocal benefit for participating communities.

Therefore, equitable participation is not just a recruitment challenge, it is a design and governance challenge.

### 2.2. Data Donation: Decentralize Participation in Cardiovascular Research

Traditionally, researchers design and conduct studies independently and reach out to participants without involvement or input of the populations that are being studied [42]. A particularly challenging bias that is typically not considered in research can occur when biobanks collect data from individuals that are not representative of their target population [43,44]. Data donation reverses this unidirectional model by enabling participants to proactively contribute to the scientific commons [45]. Participating individuals take an active role in forming the research ecosystem, rather than being passive research subjects. In practice, this requires clear governance structures, transparent data-access policies, and consent models that allow participants to retain agency over how their data are used over time.

The concept of data donation is gaining attention across Europe and North America, sourced by increasing public interest health data, data privacy, digital health, and the desire to contribute to societal good [46,47].

Data donation has been described as a form of citizen science by Bietz et al., where individuals contribute their electronic health records, wearable data, or genomic information to health research initiatives [48]. While their work primarily outlines the framework and values behind such efforts, such as focusing on trust, governance, inclusivity, and feedback mechanisms, the paper also highlights the design of emerging donation models.

One of today’s most fully realized examples of data donation is the UK Personal Genome Project (PGP-UK). A model of radical transparency invites volunteers to donate their genomic, health, and lifestyle data to open access repositories. Participants provide explicit consent for the data to be made publicly available. This also includes acceptance of potential reidentification risks. The project demonstrates how voluntary openness can advance both science and citizen empowerment, as long as it is well-governed and communicated [47].

In Germany, over half a million people participated in the *Corona-Datenspende App.* This voluntary initiative, which was launched during the COVID-19 pandemic, collected anonymized data from wearables (e.g., heart rate, temperature) to support infection surveillance. While this app was time-limited and not open access, it showed that a significant portion of the public is willing to share personal health data when the purpose is clear and societally relevant [49].

Various stakeholders in Germany are further exploring the potential of secure digital platforms to enable citizens to manage and potentially share their health data in the future [50]. One of these platforms is *Meine Gesundheit*. Although this is not yet a formal data donation model, these efforts signal growing institutional interest in ethically governed, citizen-centered data infrastructure. The model aims at building public trust through clear consent procedures, accessible information, and tangible feedback, such as summaries of collective findings.

In the Netherlands, early concepts like *TRAIN Health Awareness* and *Doneer je Data* emphasize the power of citizen-led science by aiming to integrate data donation into daily life [46,51]. The *TRAIN Health Awareness* platform uses existing digital channels, such as fitness wearables or hospital patient portals, to invite people to share data on cardiovascular risk factors and lifestyle. By lowering barriers to participation and integrating recruitment into commonly used digital platforms, such as patient portals or fitness apps, this model can generate more diverse and representative data than traditional cohort studies [46]. One of the main goals is to expand the cardiovascular research base by ensuring that populations that are often left out of studies, such as people with less education, lower income, or long-term health conditions, are included and that their experiences are reflected in the data. The recently launched *TRAIN India* initiative extends this model to a diverse, multi-ethnic population across urban and rural regions, building local partnerships with clinicians, health influencers, and community organizations. This cross-sectoral approach is developed not only to increase representation, but to co-generate insights that are locally grounded and globally relevant. In this way, TRAIN offers a blueprint for how digital innovation can advance data equity beyond the scope of conventional cohort studies and align with emerging approaches in citizen-led and decentralized research.

An inclusive design is essential for a successful data donation system. Interfaces should be multilingual, readable at different literacy levels, and accessible to people with disabilities. Transparency should lead to in an increase in trust among the participants: who can access the data, for what purpose, and under what safeguards? Dynamic consent models allow participants to maintain agency over time, updating their preferences as their comfort or circumstances evolve. Ethics committees and regulators need to develop criteria that explicitly address participant-driven consent, shared data governance, and accountability in decentralized research models [48].

Data donation may also offer opportunities in low-resource settings, where formal research infrastructure is limited. While large-scale projects are rare, pilot studies in community networks (e.g., mobile health surveys or decentralized biometric data capture) have explored how voluntary participation, when grounded in trust and cultural sensitivity, can create locally relevant datasets. However, these examples remain nascent and require further ethical and technical development before scaling.

At the same time, these approaches face important practical constraints, particularly in LMICs. Regulatory frameworks differ across countries and institutions. Limited digital infrastructure, fragmented health information systems, and varying data protection regulations can complicate large-scale implementation. This can complicate data sharing and governance. Combining data from multiple sources raises ongoing challenges related to data quality and interoperability. In addition, participatory and globally coordinated models require sustained funding, infrastructure, and expertise, which are not equally available across settings. Ethical oversight may also be challenged by differing consent norms and governance traditions, while long-term sustainability depends on stable funding and local capacity building rather than short-term pilot financing.

**Figure 2:**
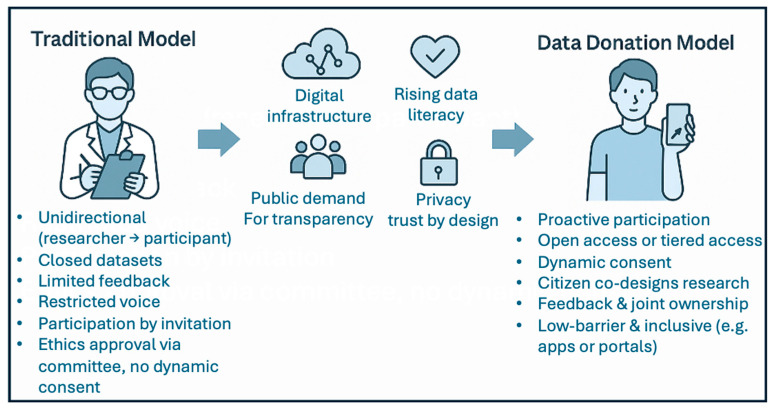
Legend: rethinking research participation: the transition to data donation models. This figure contrasts traditional researcher-centric models of data collection with emerging data-donation approaches. These models emphasize voluntary participation, dynamic consent, and ongoing engagement between participants and researchers.

Importantly, data donation is not a replacement for traditional cohort studies, but a complement. By enabling people to contribute in flexible, user-defined ways, it expands cardiovascular research beyond academic hospitals and into real-world settings, offering a richer, more representative understanding of health and disease (Figure 2).

### 2.3. Global Data Infrastructures: Fair Representation in Knowledge Generation

The challenges of representation of participants in scientific projects and datasets are not confined to recruitment, but extend into data access and harmonization. Global disparities in infrastructure and governance often hinder collaboration, leading to fragmented silos of valuable but underutilized data [52,53]. Ethnicity is not always registered in population studies and patient files. Furthermore, ethnicity is registered in different ways in different countries. This lack of interoperability obstructs large-scale analyses needed to understand for instance region-specific disease patterns or gene–environment interactions. Calls for arrangements that facilitate collaborative research have led to global funding schemes and programs that seek to leverage international partnerships for environmental change research. An increasing number of papers, however, indicate that new science institutions for sustainability are shaped by internal divisions and inequalities preventing them from realizing their ambition [54].

In recent times, a few models have shown some potential. *The Global Alliance for Genomics and Health (GA4GH)* has developed data-sharing frameworks that respect local sovereignty while enabling global research [55]. The *100,000 Genomes Project* [56] in the UK and the *All of Us Research Program* [57] in the US include ethnic and geographic diversity as core design elements, while creating open platforms for international collaboration.

The *H3Africa Initiative* has successfully built genomics capacity across the continent in Africa [58]. Its cardiometabolic studies have already revealed unique genotype–phenotype correlations in populations that have historically been excluded from global genomic science [59]. Similar efforts are emerging in Southeast Asia, Latin America, and the Middle East. However, these initiatives require long-term investment in digital infrastructure, research training, and ethical governance.

New technologies such as federated learning offer promising tools for ethical and cross-border research, as algorithms are trained across decentralized datasets [60]. These systems protect local ownership while enabling pattern discovery at scale. However, they require significant coordination and trust frameworks to function responsibly.

Bridging global data also means respecting pluralism in health knowledge. In many regions, traditional medicine coexists with biomedicine and understanding how cardiovascular risk is perceived and managed requires cultural as well as clinical insight. Integrating such knowledge systems can make global cardiovascular science not only more inclusive, but also more effective.

## 3. From Data to Action: Rebuilding Guidelines, Risk Models, and Tools

Representation of diverse communities in data is crucial; however, it is only the first step. For cardiovascular care to become truly equitable, we must redesign the tools, algorithms, and clinical frameworks that transform data into clinical decisions.

### 3.1. Methodological Limitations in Current Cardiovascular Risk Prediction Models

Cardiovascular risk prediction tools are foundational to modern cardiovascular medicine. Yet the most widely used models, such as the Framingham Risk Score [61] or the Pooled Cohort Equations [62], were built on narrow, homogeneous datasets. Their performance drops significantly in individuals from under-represented backgrounds, leading to both under-treatment and over-treatment [63,64].

For instance, the Framingham model systematically fails to predict cardiovascular risk in under-represented groups. Among South Asian people [65], particularly those from socioeconomically deprived areas [66], the risk is generally underestimated, while overestimated risk scores are more common among African [67,68] and East Asian populations [69]. In Indigenous populations in Canada and Australia, where CVD tends to present earlier and with different comorbidity profiles [70,71], standard risk models miss high-risk individuals entirely [72]. These misclassifications are not just statistical; they affect real decisions about statin use, blood pressure thresholds, or even candidacy for invasive procedures [73].

Moreover, newer models incorporating polygenic risk scores (PRSs), which are promising in high-income European ancestry groups, amplify this inequity [74]. Because most genome-wide association (GWAS) data come from Northern European populations, the predictive power of PRS declines steeply in individuals of African [75], Asian [76], or admixed ancestry [77,78,79]. This has led to growing concern that the next generation of precision medicine could widen existing health disparities unless action is taken.

Emerging efforts are attempting to recalibrate these models [80,81]. Initiatives like the *Multi-Ethnic Study of Atherosclerosis (MESA)* [82] and the *UK Biobank’s pan-ancestry analyses* [83] have begun to produce more diverse and inclusive risk estimates. In South Africa, the *Birth to Twenty Plus* cohort, which is the largest and longest-running longitudinal birth cohort in Africa, offers a uniquely valuable dataset for building regionally relevant tools [84,85,86]. Meanwhile, the *Jackson Heart Study* in the U.S. is currently enabling recalibrated risk scores for African American populations [87]. Furthermore, the China-PAR model tailors risk predictions to dietary, behavioral, and epidemiological profiles specific to Chinese adults [88]. However, locally validated models remain the exception not the rule, particularly in regions like Latin America [89] or in the Pacific Islands, where cardiovascular burden is rising but data infrastructures are limited [90].

Inclusive risk models require representative input, contextual calibration, and ongoing validation across populations, along with a clear regulatory and ethical framework to prevent algorithmic bias. If this is not implemented, well-intentioned innovation risks reinforcing the very inequities it aims to solve.

### 3.2. Guidelines: From Universality to Contextual Intelligence

Evidence-based clinical practice guidelines have the potential to reduce health inequities and improve care among disadvantaged populations [91]. However, guidelines can also unintentionally create or exacerbate existing health inequities between populations [92]. Clinical guidelines form the foundation of cardiovascular care, but their “one-size-fits-all” design often fails to account for population-specific variation [93]. For example, current aortic surgery thresholds (such as a 5.5 cm cutoff for thoracic aortic aneurysm surgery [14]) are based primarily on data from White male cohorts [94,95,96]. Yet aortic rupture and dissection can occur at smaller diameters in women and in individuals with certain genetic backgrounds or smaller body sizes, such as people of East Asian ancestry [97]. Applying uniform thresholds to diverse bodies can lead to delayed surgery and worse outcomes.

Hypertension guidelines are based on Western populations and therefore might not translate well to countries where the pathophysiology of hypertension is different [98], salt sensitivity is higher [99], or access to lifelong medication is less reliable [100]. In South and Southeast Asia, for example, individuals often experience higher rates of stroke and left ventricular hypertrophy at lower blood pressure thresholds [101]. Yet local data are often missing from the evidence base, and guidelines are still based on extrapolation rather than direct evidence.

A growing number of professional societies are beginning to issue adapted or region-specific guidelines, such as the World Heart Federation’s framework for low-resource settings [102], or the Indian Council of Medical Research’s ethnic-specific risk scores [103]. In Canada, community-based screening programs have challenged national CVD screening criteria and advocated for earlier, culturally adapted intervention protocols [104].

The WHO’s PEN (Package of Essential Noncommunicable Disease Interventions) program further illustrates this shift. In countries such as Nepal and Ethiopia, where formal infrastructure is lacking, simplified paper-based risk stratification tools enable community health workers to triage cardiovascular risk [105]. These adaptations underscore how structural constraints must inform what is clinically actionable. Incorporating health equity into clinical practice guidelines remains a challenge because there is no widely accepted guidance or standard for reporting quality and the few available tools or checklists for evaluating guideline quality do not include health equity [106]. We need to shift from rigid universality toward contextual intelligence, guidelines that are adaptive, evidence-based, and sensitive to social determinants, genetics, gender, environment, and health system constraints.

### 3.3. Tools for Designing for Equity

Advances in genomics, AI, and digital health technologies have powered the development of precision medicine, offering new paths for targeted therapies. However, challenges remain, including the under-representation of diverse populations in research and the need to update clinical reference intervals to address the unique needs of resource-limited populations and the importance of global diversity in precision medicine strategies. Risk scores and guidelines may be developed by academics, but they are implemented by clinicians all over the world. These clinical decisions are often taken under time pressure, with incomplete data, and in systems with limited resources. Designing decision-support tools that reflect real-world diversity is therefore crucial.

For instance, in many parts of the world, mobile health (mHealth) platforms are becoming the primary interface for both patients and providers. In rural Kenya, the *AFYACHAT* tool uses SMS-based triage and risk scoring for hypertension [107]. In Canadian community clinics, nurses use tablet-based algorithms to screen for cardiovascular risk in underserved areas. These digital tools are not only adaptable and low-cost, but they also demonstrate how equity-focused design can bridge resource gaps [108].

Digital tools also allow for dynamic, personalized risk assessment, combining local epidemiology with patient-specific data. However, equitable design must go beyond software: language, literacy, gender sensitivity, and cultural usability all play a role in whether tools are actually adopted [109].

Meanwhile, clinical AI systems, which are becoming increasingly prevalent in hospital settings, must be held to higher standards of transparency and fairness. Several studies have shown that Black patients are systematically deprioritized in AI-driven triage systems trained on biased Western datasets [110]. However, equity-oriented tools can fail when implementation is weak. Algorithms that are recalibrated without validation across populations may introduce bias. Participatory research does not change power relations if community involvement is limited or symbolic. Clear evaluation, reporting by subgroup, and defined responsibility remain necessary. To prevent these failures, algorithms must be tested explicitly for disparate performance and adjusted accordingly. This includes open reporting of performance across subgroups, community co-design, clear regulatory pathways and broader inclusion of participants.

Finally, principles of data sovereignty, which are particularly relevant for Indigenous and marginalized populations, should also govern how digital tools are implemented. As seen in Canada and New Zealand, community-led control over how health data are used can improve both trust and tool effectiveness [111].

In essence, equity cannot be a post hoc consideration. It must be a design principle from the start.

## 4. Changing the System: Structural Levers for Equitable Cardiovascular Health

To transform cardiovascular care, it is essential to sustain equity. In addition to better data or smarter tools, the systems that govern how science and care is financed, delivered, and valued require attention. Communities most affected by CVD should have a voice in shaping their own health futures. To achieve this, systemic biases need to be confronted and incentive structures rethought.

### 4.1. Financing Models That Reward Prevention and Equity

Most health systems remain structured around acute interventions, surgeries, and hospital admissions [112]. This fee-for-service model inherently disincentivizes equity-driven strategies that could reduce CVD burden most efficiently. Early risk detection, community-based care, and social support interventions receive less attention.

Capitation-based systems, bundled payments, and value-based care initiatives could offer a potential path forward. *Family Health Strategy* is an initiative in Brazil in which teams of community health workers deliver preventive care at scale in low-income neighborhoods. This practice is supported by a financing model that rewards population-level outcomes rather than procedures [113]. In Rwanda, rural clinics use performance-based financing incentivize hypertension screening. This approach has led to significant improvements in detection and treatment adherence [114].

Yet even value-based care can cause disparities if not carefully designed. If performance metrics do not adjust for socioeconomic complexity or access barriers, providers serving marginalized populations may be penalized rather than rewarded. Equity-adjusted payment models, such as those piloted in the United States’ Medicaid program, explicitly account for social risk in their reimbursement formulas and are beginning to show promise [115].

From an economic perspective, implementing more inclusive data infrastructures and equity-oriented care models requires investments. And to support equity, financing mechanisms must evolve from a moral imperative to a measurable, fundable outcome. The costs of inclusive science need to be weighed against the potential long-term benefits. As investing in equity can support more accurate risk stratification, reduced overtreatment or delayed diagnosis, and better allocation of limited healthcare resources.

### 4.2. Workforce, Representation, and Cultural Competence

Besides access, equity in cardiovascular care depends on a workforce that reflects the communities it serves and is equipped with cultural competence. Despite growing awareness, structural barriers continue to limit diversity throughout the training pipeline [116,117]. Many medical education programs lack formal curricula on social determinants of health, implicit bias, and the unique cardiovascular risks which are faced by non-European populations.

Studies have shown that racial and ethnic concordance between patients and providers improves adherence, trust, and satisfaction, particularly in preventive care [118,119]. Yet in many countries cardiology remains among the least diverse specialties [120]. In the U.S. for example, Black and Hispanic physicians make up less than 10% of the cardiology workforce, despite serving a population of which nearly 40% identify as non-White [121].

Beyond representation, training must equip all providers with the tools to recognize structural drivers of cardiovascular risk. Programs such as South Africa’s Ukwanda Rural Clinical School or the UK’s Widening Participation initiative have shown that recruiting students from underserved communities can both diversify the workforce and improve retention in high-need areas [122,123].

Moreover, culturally tailored interventions which are delivered by trusted community members can often outperform traditional models [124,125]. Faith-based hypertension screening campaigns led by religious leaders in mosques and churches significantly increased treatment uptake [126,127,128]. Culturally rooted walking programs have also demonstrated measurable reductions in cardiovascular risk markers worldwide [129].

### 4.3. Governance, Inclusion, and Health Sovereignty

The diverse range of diseases, risk factors, healthcare access, health equity, and geographical characteristics all contribute to the uniqueness and variability of health problems within a population. Many national cardiovascular strategies are developed without meaningful input from the communities which are most affected. Over the years, this has resulted in policies that are technically sound but socially misaligned. Achieving equity in cardiovascular care therefore requires that communities are meaningfully involved in agenda-setting, decision-making, and evaluation, rather than being consulted only after policies or programs have been designed.

At a policy level, the abovementioned principles can be operationalized through concrete regulatory and funding mechanisms. Examples include mandatory disaggregated reporting by sex, ethnicity, and geography in publicly funded cardiovascular research; equity criteria embedded in grant calls and reimbursement models; and requirements for local co-leadership and shared governance in international research collaborations. At an international level, alignment with existing frameworks such as GA4GH data-sharing standards or Indigenous data sovereignty principles can support enforceability while respecting local autonomy.

Local demographic and healthcare infrastructure factors significantly influence CVD outcomes [130]. Recent studies highlighted the importance of place-based interventions in addressing cardiovascular health disparities [131]. Therefore, community advisory boards, citizen panels, and participatory policymaking models offer promising alternatives. In Canada, the First Nations Health Authority operates with full governance control over health services for Indigenous populations, integrating traditional healing with Western medical care [132]. In Colombia, Afro-Colombian communities in Chocó (e.g., Quibdó) have engaged in participatory partnerships with universities to co-design hypertension prevention programs tailored to regional disease profiles and cultural practices, demonstrating significant reductions in blood pressure following community-led implementation [133].

Data governance is also central. For too long, health data have been extracted from marginalized communities without transparency, reciprocity, or benefit-sharing. Ethical frameworks such as *OCAP (Ownership, Control, Access, and Possession)* developed by Canadian First Nations [134], or *Te Mana Raraunga* in Aotearoa (New Zealand) [135], emphasize Indigenous data sovereignty and self-determination. These models are increasingly being adopted as standards for research and health systems [136].

Inclusive governance also applies to the global level. Many LMICs remain under-represented in the leadership of major cardiovascular societies, despite bearing the highest burden of disease [137]. Funding agencies and academic journals have a role to play by mandating local co-leadership in global health projects and ensuring authorship equity in publications.

## 5. Call to Action: From Representation to Reparation

Cardiovascular medicine is advancing rapidly, ranging from AI-assisted diagnostics to gene-guided risk stratification and treatment options [138,139]. The priorities outlined below are grounded in the evidence discussed throughout this review, which consistently shows how lack of diversity in data and governance affects risk prediction, guideline validity, and clinical outcomes. However, even the most sophisticated novel tools can reinforce inequity if they are built upon narrow datasets, guided by biased algorithms, or implemented in systems that systematically exclude certain demographics. Innovation, in isolation, is not inherently just.

As this paper has shown, equity in cardiovascular science requires far more than diversifying participant pools. It requires reimagining the systems that produce, apply, and regulate knowledge, from research design to reimbursement models, from clinical guidelines to digital tools. At its core, this is not simply about inclusion but about reparation: correcting a long-standing imbalance in whose data is valued, whose needs are prioritized, and whose lives are saved.

### 5.1. From Data Inclusion to Structural Change

This roadmap should be read as a sequence rather than a single intervention. Data inclusion and governance come first, followed by translation into models, guidelines, and tools. Broader reforms in financing, workforce, and policy are required to sustain these efforts.

Epidemiological data is a valuable resource to support decision-making in a clinical setting. It not only serves direct purposes by supporting evidence-based treatment, but also indirectly contributes to guidelines and policies in healthcare services. It is common for us to depend on epidemiological data from foreign countries, often HICs, but this practice can introduce bias into decision-making process due to the disparities between their conditions. Efforts to diversify data, whether through community-based research, global genomic cohorts, or data donation platforms, are vital first steps [140]. But paying attention to inclusion alone is insufficient. The resulting insights should be translated into care that is accessible, affordable, and aligned with the lived realities of diverse populations. Representation in datasets must be matched by representation in authorship, leadership, and agenda-setting. Structural inequities require structural solutions.

For instance, including Indigenous populations in risk models is important, but so is ensuring that care is delivered through culturally safe services, supported by Indigenous health workers, and embedded in community-led governance. Similarly, building polygenic risk tools for African populations means nothing if those tools are not paired with health infrastructure that can act on the risk.

### 5.2. The Role of Institutions and Accountability

Academic institutions, funders, journals, and regulators play a central role in shaping incentives and standards, and therefore have a responsibility to support more equitable cardiovascular research and care. Equity impact assessments should be embedded in grant reviews and protocol approvals. Such requirements could be formalized through national research funding legislation, conditional public funding, and harmonized international research agreements that mandate local partnership and equitable data governance. Peer reviewed journals should mandate disaggregated reporting by sex, ethnicity, and geography, not as supplementary tables but as central findings. Ethics committees must adapt to new models of data ownership and community consent, and adaptation is becoming especially essential as decentralized and participant-driven research models grow.

Abovementioned priorities must also become central in the training of the next generation of cardiovascular professionals. Equity should no longer be treated as a peripheral consideration in cardiovascular research, training, and practice, but should be a core competency. Medical curricula should include structural determinants of health, cultural safety, and bias in clinical reasoning and improve the methods used for the selection and recruitment of diverse students and specialists. Leadership in cardiology must open up space for diverse voices, particularly from under-represented geographies and disciplines.

### 5.3. Shared Ownership of the Future

Cardiovascular health is deeply shaped by social, political, and economic forces and not merely a medical outcome. Therefore, the responsibility for achieving equity must be collective. Taken together, the evidence reviewed in this paper points to a set of actionable priorities across research, clinical practice, policy, and education.

- Clinicians should be supported to critically assess default guidelines and advocate for context-sensitive care.
- Researchers and clinicians should find more ways to enrich data, e.g., by registering ethnicity in patient files and including social determinants of health.
- Researchers should focus more on co-creating studies with the communities they serve and disaggregating findings to reveal, not obscure, disparities.
- Technology experts should ensure that algorithms are applicable to diverse populations and should be transparent about their limitations.
- Policymakers need to be supported to fund prevention equitably, reform perverse incentives, and align regulatory frameworks with real-world needs.
- Communities should have opportunities for empowerment not only as participants, but as co-developers of cardiovascular science projects.
- Medical schools need to be supported to address under-representation of students and specialists from minority groups and take measures to integrate competencies in their curricula to support students to deliver diversity sensitive healthcare.

## 6. Conclusions

Addressing disparities in cardiovascular care is essential for improving outcomes and achieving health equity for all patients. Health-related disparities are driven by complex factors, including social determinants of health, gender, ethnicity and limited access to high-quality care. Targeted interventions, such as risk-based screening guidelines, culturally competent care, community-based initiatives, and policy reforms, could reduce these inequities. Prioritizing inclusivity and implementing personalized treatment strategies can help bridge gaps in care.

## Data Availability

No new data were created or analyzed in this study.

## Abbreviations

The following abbreviations are used in this manuscript:

CVD	Cardiovascular disease.
LMICs	Low- and middle-income countries.
HICs	High-income countries.
CBPR	Community-based participatory research.
PRS	Polygenic risk scores.
GWAS	Genome-wide association studies.
mHealth	Mobile health.
