# Correction: Grewal et al. Diversity and Representation in Cardiovascular Research: Evidence Gaps, Emerging Models, and Policy Implications. *Int. J. Environ. Res. Public Health* 2026, *23*, 241

**DOI:** 10.3390/ijerph23060803

**Published:** 2026-06-16

**Authors:** Simran Grewal, James Wildish, Catherine Chalmers, Christine Dedding, Jeanine Suurmond, Charles Agyemang, Nimrat Grewal

**Affiliations:** 1 Shoulder and Elbow Unit, Department of Orthopaedic Surgery, Onze Lieve Vrouwe Gasthuis, 1091 AC Amsterdam, The Netherlands; s.k.grewal@olvg.nl; 2 Department of Research, The Original Fit Factory, Glasgow G62 7LW, UK; james@theoriginalfitfactory.com (J.W.); catherine@theoriginalfitfactory.com (C.C.); 3 Department of Ethics, Law & Humanities, F-Vleugel Medische Faculteit, Amsterdam UMC, De Boelelaan 1089a, 1081 HV Amsterdam, The Netherlands; c.dedding@amsterdamumc.nl; 4 Department of Primary and Community Care, Radboudumc, 6525 GA Nijmegen, The Netherlands; 5 Department of Public and Occupational Health, Amsterdam Public Health, University of Amsterdam Medical Centers, 1105 AZ Amsterdam, The Netherlands; c.o.agyemang@amsterdamumc.nl; 6 Division of Endocrinology, Diabetes, and Metabolism, Department of Medicine, Johns Hopkins University School of Medicine, Baltimore, MD 21205, USA; 7 Department of Cardiothoracic Surgery, Amsterdam University Medical Center, Location AMC, 1105 AZ Amsterdam, The Netherlands; 8 Department of Cardiothoracic Surgery, Yale New Haven Hospital, Yale University School of Medicine, New Haven, CT 06510, USA

**Missing Conflict of Interest**

In the original publication [1], the Conflict of Interest declaration did not fully reflect the required disclosure of conflicts of interest. The corrected Conflict of Interest appear below. The authors state that the scientific conclusions are unaffected. This correction was approved by the Academic Editor. The original publication has also been updated.
